# Effects of linguistic context and noise type on speech comprehension

**DOI:** 10.3389/fpsyg.2024.1345619

**Published:** 2024-02-05

**Authors:** Laura P. Fitzgerald, Gayle DeDe, Jing Shen

**Affiliations:** ^1^Speech Perception and Cognition Laboratory, Department of Communication Sciences and Disorders, College of Public Health, Temple University, Philadelphia, PA, United States; ^2^Speech, Language, and Brain Laboratory, Department of Communication Sciences and Disorders, College of Public Health, Temple University, Philadelphia, PA, United States

**Keywords:** speech comprehension, linguistic context, speech in noise, acoustic challenges, task-evoked pupil response, speech perception, perceptual complexity, linguistic complexity

## Abstract

**Introduction:**

Understanding speech in background noise is an effortful endeavor. When acoustic challenges arise, linguistic context may help us fill in perceptual gaps. However, more knowledge is needed regarding how different types of background noise affect our ability to construct meaning from perceptually complex speech input. Additionally, there is limited evidence regarding whether perceptual complexity (e.g., informational masking) and linguistic complexity (e.g., occurrence of contextually incongruous words) interact during processing of speech material that is longer and more complex than a single sentence. Our first research objective was to determine whether comprehension of spoken sentence pairs is impacted by the informational masking from a speech masker. Our second objective was to identify whether there is an interaction between perceptual and linguistic complexity during speech processing.

**Methods:**

We used multiple measures including comprehension accuracy, reaction time, and processing effort (as indicated by task-evoked pupil response), making comparisons across three different levels of linguistic complexity in two different noise conditions. Context conditions varied by final word, with each sentence pair ending with an expected exemplar (EE), within-category violation (WV), or between-category violation (BV). Forty young adults with typical hearing performed a speech comprehension in noise task over three visits. Each participant heard sentence pairs presented in either multi-talker babble or spectrally shaped steady-state noise (SSN), with the same noise condition across all three visits.

**Results:**

We observed an effect of context but not noise on accuracy. Further, we observed an interaction of noise and context in peak pupil dilation data. Specifically, the context effect was modulated by noise type: context facilitated processing only in the more perceptually complex babble noise condition.

**Discussion:**

These findings suggest that when perceptual complexity arises, listeners make use of the linguistic context to facilitate comprehension of speech obscured by background noise. Our results extend existing accounts of speech processing in noise by demonstrating how perceptual and linguistic complexity affect our ability to engage in higher-level processes, such as construction of meaning from speech segments that are longer than a single sentence.

## 1 Introduction

Speech perception is a difficult and complex task in naturalistic settings. Variables related to the speaker (e.g., accent), the listener (e.g., familiarity with the topic), and the environment (e.g., background noise) all influence the degree of difficulty that a listener may experience in perceiving and interpreting what they hear (Freyman et al., 2004; Kidd et al., 2005; Desjardins and Doherty, 2013; Buss et al., 2022). As a result, speech perception draws on perceptual, linguistic, and cognitive processes to support recognition and interpretation of the speech stream. When one of these processes becomes heavily taxed, we draw upon others to fill in the blanks. For example, when perceptual challenges such as background noise arise, factors such as linguistic context and prior knowledge can support comprehension. For this reason, it is critical that researchers and clinicians understand how these different factors interact during speech perception. However, audiologic assessments used in both clinical and research settings largely do not incorporate linguistic context, resulting in a lack of understanding of how perceptual and linguistic factors interact during real-world speech perception in noise. Additionally, these assessments typically require repetition rather than interpretation of speech material. In order to develop more ecologically valid audiologic assessments, a greater understanding of how speech perception unfolds in naturalistic contexts with perceptual challenges and pragmatic constraints is first needed. The current study aimed to illuminate mechanisms underlying speech perception in noise by examining the interaction between perceptual challenges from noise and linguistic context during speech comprehension.

The type of noise is a frequently studied variable that makes speech perception challenging. Background noise can hinder or disrupt speech perception through energetic masking, informational masking, or both (Mattys et al., 2009). Energetic masking may occur when spectral (i.e., frequency-related) and temporal (i.e., time-related) features overlap across the target speech and the background noise, while shared linguistic features such as lexical and/or acoustic-phonetic similarity may lead to informational masking of the target speech (Kidd and Colburn, 2017). Much of the literature examining speech perception in noise has compared energetic masking to informational masking by comparing the effects of presenting speech in steady-state noise (SSN) versus using intelligible speech maskers (e.g., Festen and Plomp, 1990; Freyman et al., 1999; Brungart, 2001; Li et al., 2004; Helfer and Freyman, 2008; McCreery et al., 2020). Further research has demonstrated that even unintelligible speech-like maskers, such as reversed speech or multi-talker babble noise, lead to informational masking (e.g., Hoen et al., 2007; Rosen et al., 2013). Comparisons across multiple types of background noise have demonstrated that intelligible speech-on-speech masking and unintelligible multi-talker babble pose a greater perceptual challenge than SSN due to the combination of informational and energetic masking (e.g., Freyman et al., 2004; Desjardins and Doherty, 2013; Van Engan et al., 2014; Wendt et al., 2018). The perceptual similarity between the acoustic-phonetic features of speech or speech-like maskers and the target speech leads to greater difficulty with speech stream segregation (i.e., separating the target speech from the masker).

Across this body of research comparing different noise types, intelligibility has been the primary means of measuring masking effects. Performance on intelligibility tasks, however, may be more reflective of working memory capacity than comprehension (Beechey, 2022). Comprehension, on the other hand, requires greater depth of processing to interpret the meaning of an entire utterance, which is the ultimate goal in real-world communication (Fontan et al., 2015). Comprehension tasks, therefore, may motivate participants to engage with and attend to the speech materials as a whole, and attention has been shown to play a role in speech comprehension (Bhandari et al., 2022). Further, there is evidence that effects of noise on speech perception differ based on the processing depth associated with different tasks (e.g., Shen et al., 2022). For these reasons, identifying how relative perceptual challenges associated with babble and SSN affect the comprehension of utterances, beyond memorization and verbatim repetition, is critical for strengthening the ecological validity of clinical tools.

Comprehension questions can be used to measure how perception challenges affect interpretation of speech material. However, they do not reflect the real-time effects of informational masking on comprehension. Task-evoked pupil dilation is a physiological measure that provides a more nuanced view of processing effort. Changes in pupil dilation, which are involuntary central nervous system responses that occur during cognitive processes (Beatty, 1982), can reflect the effort a listener is expending during online speech processing (Burlingham et al., 2022). Given that speech processing occurs continuously as an utterance unfolds (e.g., Norris and McQueen, 2008; Wendt et al., 2014; Bentum et al., 2019), processing effort (as indicated by pupil dilation) coupled with behavioral assessments of speech comprehension provides a more fine-grained measure of how speech processing is affected by the informational masking from babble noise. Aligned with previous literature that has used peak pupil dilation (PPD) to index processing effort (e.g., Winn et al., 2015; Wendt et al., 2018; Shen et al., 2022), we measured changes in pupil dilation during a time window surrounding the final word of each stimulus (which differed across context conditions) to gain insight into speech comprehension in noise as processing unfolds.

The present study first examined whether noise type has a differential effect on performance and processing effort during a speech comprehension task. That is, will the acoustic-phonetic interference associated with multi-talker babble noise lead to poorer comprehension performance compared to when speech is presented in spectrally matched SSN? To answer this question, we compared behavioral (i.e., accuracy and reaction time when answering comprehension questions) and physiological (i.e., pupillometric) responses to speech material presented in either unintelligible multi-talker babble noise or in spectrally matched SSN. If acoustic-phonetic interference affects the comprehension of spoken input, we would expect to see lower accuracy (indicating poorer comprehension), slower reaction times, and greater pupil dilation (reflecting increased listening effort) for speech stimuli presented in multi-talker babble as compared to SSN. If, on the other hand, there is no effect of acoustic-phonetic interference on comprehension, we would expect to see little difference in accuracy, reaction time, and pupil dilation between the two noise conditions. Assessing both comprehension accuracy and reaction time (offline, post-stimulus measures) and listening effort (an online measure occurring in real-time throughout speech processing) constitutes a novel approach to examining the perceptual effect of noise in speech processing. Incorporating both offline and online measures of speech comprehension is more ecologically valid than measuring intelligibility alone, which is the typical approach in much of the extant hearing science literature.

Acoustic input is just one component of the complex, dynamic system that is verbal communication (Larsen-Freeman, 1997). Listeners may rely on other sources of information available to them to compensate for the perceptual ambiguity caused by informational masking. One such source is linguistic context. In a much-cited study introducing the “cloze” procedure, Taylor (1953) demonstrated that listeners can reliably fill in an omitted word from a sentence based on the surrounding words. This procedure was originally designed as a text readability measure, but it has since been applied in speech perception studies. For example, Wingfield et al. (1991) found that participants required less acoustic information to identify words in a recognition task when target words were embedded in a sentence providing linguistic context versus in isolation. More recent research following this line of inquiry has examined how linguistic context can help listeners fill in perceptual gaps created by acoustic challenges. Van Os et al. (2021) used an adaptation of Taylor’s cloze procedure with recorded sentences presented in multi-talker babble noise. Participants repeated the final word of sentences that either ended in a target word that fit the linguistic context or a distractor word that was phonetically similar to the target word but did not fit the context of the sentence. In quiet, participants correctly repeated the final words, regardless of whether a target or an incongruent distractor was presented. However, with multi-talker babble noise added, participants tended to produce a word that fit the linguistic context, even in the distractor condition. These findings suggest that listeners rely on the acoustic signal when acoustic challenges are minimal but use linguistic context to resolve perceptual ambiguity when acoustic challenges arise.

Studies using the cloze task are consistent with the Ease of Language Understanding model (ELU; Rönnberg et al., 2013), which outlines the process of word recognition when listening to acoustically degraded speech. Importantly, however, the ELU does not provide an account for the effects of effortful word recognition that could manifest at higher levels of processing (i.e., at sentence or discourse level). This knowledge gap is also reflected by the fact that the effect of linguistic context in hearing research has typically been measured by recognition of sentence-final words (e.g., Bilger et al., 1984; Pichora-Fuller et al., 1995). Although this body of evidence demonstrates that listeners use sentence context during speech perception in noise, word recognition does not require the same depth of processing needed for speech comprehension, and therefore falls short in demonstrating the interactive effects of context and noise on speech processing. Thus, the second research question we examined in the present study relates to the interaction between perceptual challenges and linguistic context during comprehension of complex speech material.

In the psycholinguistic literature, current theories of spoken sentence processing postulate that listeners generate predictions about upcoming speech material in real time to maximize the efficiency of sentence comprehension (Hale, 2001; Levy, 2008; Gibson et al., 2019). Prior studies have examined how linguistic context influences sentence processing using physiological data and comprehension questions. However, these studies typically focus on reading comprehension (e.g., Just and Carpenter, 1993; Scharinger et al., 2015; Fernández et al., 2016). In studies of auditory comprehension (e.g., Engelhardt et al., 2010; Piquado et al., 2010; Wendt et al., 2016; Ayasse et al., 2021), the linguistic manipulation has primarily been syntactically focused (e.g., passive constructions, embedded relative clauses, and garden path sentences) and/or related to plausibility. These manipulations typically result in sentence structures that are unlikely to be heard in everyday conversational contexts. Moreover, unrelated sentences were presented in isolation, absent of any context and without background noise (with the exception of Wendt et al., 2016), which limits the ecological validity of the studies. In the current study, we used sentence pairs to provide more context and presented stimuli in background noise, two factors that are commonly present in real-world conversational contexts. These sentence pairs provide descriptions of scenarios involving agents with goals, cause and effect, and a timeline of events. These factors are used to build situation models of language input (Zwaan, 1996; Zwaan and Radvansky, 1998). As a result, these speech materials allowed us to examine the impact of informational masking on processing of longer and more semantically complex speech material.

There is relatively little empirical evidence demonstrating how background noise affects listeners’ ability to generate predictions based on linguistic context. Evidence from electroencephalography (EEG) suggests that background noise disrupts listeners’ ability to generate predictions: brain wave responses to unexpected semantic input (i.e., N400 effects) tend to be delayed when speech is presented in background noise (Connolly et al., 1992; Silcox and Payne, 2021; Hsin et al., 2023). On this account, listeners may be less efficient when facing greater perceptual complexity (i.e., acoustic-phonetic similarity) associated with informational masking (e.g., babble noise) as compared to energetic masking (e.g., SSN). In other words, informational masking may have a more detrimental impact on the efficiency with which listeners can engage in deeper processing and generate predictions from the linguistic context than energetic masking (*cf.* Gibson et al., 2019). If informational masking causes listeners to be less efficient in language processing, they may benefit more from supporting linguistic context to comprehend speech in babble noise than in SSN. This efficiency-oriented view would predict listeners’ greater reliance on top-down information from linguistic context in informational masking (babble) than energetic masking (SSN), leading to greater disruption when presented with a word that does not fit the context.

In contrast, another body of research suggests that perceptual complexity can hinder a listener’s ability to use top-down information from the linguistic context. This line of inquiry relates to the effortfulness hypothesis posited by Rabbitt (1991). According to this hypothesis, background noise can impact comprehension and encoding of information in memory, even when intelligibility is not affected (Rabbitt, 1991). Findings from audiology research have indicated that the effort expended to resolve perceptual ambiguity in adverse listening conditions results in reduced cognitive resources available for interpreting and making use of linguistic context, particularly for syntactically complex utterances (Wingfield et al., 2006; Pichora-Fuller et al., 2016; Peelle, 2018). In other words, perceptual difficulties may have “downstream” effects on higher levels of processing (McCoy et al., 2005). The consequences of these downstream effects, which are captured by the Framework for Understanding Effortful Listening (FUEL) (Pichora-Fuller et al., 2016), include fatigue and depletion of cognitive resources that are necessary to engage in higher-level processes such as generating predictions based on linguistic context. On this account (Rabbitt, 1991; McCoy et al., 2005; Wingfield et al., 2006; Pichora-Fuller et al., 2016; Peelle, 2018), informational masking depletes cognitive resources needed for processing information, building situation models, and generating predictions based on linguistic context. Thus, this effortfulness-oriented view would predict that listeners’ ability to make use of linguistic context may be greater in SSN than in babble.

In sum, the present study addressed two objectives. The first objective was to identify the main effects of the type of background noise (i.e., unintelligible multi-talker babble versus SSN) on comprehension performance and listening effort. The second objective was to examine how linguistic and perceptual variables (i.e., context and noise) interact during speech comprehension in noise. Building on the psycholinguistic evidence of context effects (Federmeier et al., 2002), we asked whether the effect of linguistic context interacts with the perceptual complexity of speech babble. Offline behavioral (i.e., comprehension question response time and accuracy) and online physiological (i.e., pupil dilation) measures were employed to shed light on this interaction. A paradigm with greater ecological validity than prior related studies was used to provide greater insight into how these processes unfold in naturalistic communication.

## 2 Materials and methods

The study protocol was approved by the Institutional Review Board at Temple University.

### 2.1 Participants

Forty-one younger adults (*M* age = 21.46, range 18 to 33 years) were recruited from Temple University and the surrounding community. 93% of participants identified as female, 5% as male, and 2% as nonbinary. One participant was removed from the analysis due to a technical error during testing. All participants had typical hearing (pure-tone average (PTA) across 0.5, 1, 2 k Hz < 25 dB HL) and were free of neurological, otological, developmental, speech-language, and uncorrected visual disorders by self-report. Participants spoke American English as their first language, defined as learning the language before age 6 and not primarily in a school environment. Participants were either paid or awarded course credit for their time.

### 2.2 Materials

#### 2.2.1 Speech stimuli

The stimulus set was comprised of 100 sentence pairs developed from a prior psycholinguistic study of semantic context effects (Federmeier et al., 2002). Each critical sentence was preceded by an initial sentence that served to establish context. There were three versions of each critical sentence differing with respect to the final word (cloze), which corresponded to one of the following categories: expected exemplar (EE), within-category violation (WV), between-category violation (BV). Expected exemplar refers to the word that would be predicted based on the context. Within-category violations were lexical items that belong to the same semantic category as the expected exemplar but did not fit the lexical context. Between-category violations were contextually incongruent lexical items that belonged to a different semantic category than the EE and WV. The critical word was always the final word in the second sentence in the pair. Table 1 presents an example of all three versions of a set of four sentence pairs and examples of subsequent comprehension questions. The WV category was included to provide additional information about whether participants use context in noise, as differences between the WV and BV conditions would indicate preactivation from linguistic context (Federmeier et al., 2002). The incorporation of the BV and WV conditions follows previous research (Federmeier et al., 2002) and allows for a more nuanced investigation of the online effects of semantic activation of words that do not fit the context but share semantic features with the expected cloze, as compared to a semantically unrelated word. This semantic activation could make the semantically related but contextually incongruous WV clozes easier to process than the unrelated and incongruous BV clozes. Federmeier et al. (2002) developed the stimuli so that critical lexical items appeared in all three conditions. That is, each cloze word occurred in three different sentence pairs: once as an expected exemplar, once as a within-category violation, and once as a between-category violation. The overall stimulus set was broken into twenty-five smaller sets, with four items (i.e., sentence pairs) within each set. Four different clozes appeared three times within each set so that each cloze would appear in each of the three context conditions. Word frequency and phonological neighborhood density were similar across critical lexical items (i.e., clozes) within each set of four sentence pairs. The mean range of lexical frequency for the clozes within each set was 64.67 (*SD* = 100.86; Brysbaert and New, 2009), and the mean range of phonological neighborhood density within each set was 23.4 (*SD* = 15.01; Marian et al., 2012).

**Table 1:** Sample item set with all context conditions and sample comprehension questions.

Sample item	Cloze	Comprehension question	Answer
The day before the wedding, the kitchen was just covered with frosting. Annette’s sister was responsible for making the…	*EE*	cake	Was the kitchen messy?	Y
	*WV*			
	cookies			
*BV*
toast
The little girl was happy that Santa Claus left nothing but crumbs on the plate. She decided he must have really enjoyed the…	*EE*	cookies	Was the girl angry?	N
	*WV*			
	cake			
*BV*
bagel
Chris moped around all morning when he discovered there was no cream cheese. He complained that without it he could not eat his…	*EE*	bagel	Was Chris upset?	Y
	*WV*			
	toast			
*BV*
cookies
He wanted to make his wife breakfast, but he burned piece after piece. I could not believe he was ruining even the…	*EE*	toast	Was he making dinner?	N
	*WV*			
	bagel			
*BV*
cake

Stimulus preparation involved cloze norming (Taylor, 1953), as certain probabilities may have changed since Federmeier et al. (2002) initially normed these materials (e.g., likelihood of drinking carrot juice). Norming data were collected from fifty young adults (age range 18–35 years) who were native speakers of American English. Participants were visually presented with 100 sentence pairs, with the critical word (i.e., the last word of the second sentence) omitted. The participants’ task was to type a word to complete the sentence. Responses for each item were coded. Expected exemplars had to appear in at least 40% of total participant responses, and the response rate for expected exemplars had to be at least two times higher than that of any other response, unless the response was the superordinate category to which the target word belonged (e.g., *dog* for target word *poodle*). Five sentence pairs that did not meet the norming criteria were rewritten and re-normed, resulting in a total of 100 sets of sentence pairs, with three versions within each set.

Stimuli were divided into three lists of 100 sentences that would be presented across three visits. Each sentence pair item appeared once on each list with a different cloze. Cloze types were counterbalanced across lists so that each list had an equal number of EE, WV, and BV items. Comprehension questions were created based on the content of the sentence pairs. Half of the questions related to content from the first sentence in the pair; the other half asked about the second sentence. None of the questions were directly related to the cloze, however. Built on previous work showing that the linguistic manipulation affects performance even when the participants’ attention is not directed to critical targets (e.g., Long and Prat, 2008), we purposefully did not target the cloze in the comprehension questions because we were interested in automatic processing of complex linguistic stimuli. Correct responses to comprehension questions were counterbalanced (50% yes, 50% no on each list).

Speech stimuli were recorded by a female speaker of General American English with background knowledge of phonetics and phonology. Sentences were segmented and scaled to equalize amplitude. The sentence pair durations ranged from 4,880–9,425 ms, with a mean of 7,183 ms.

#### 2.2.2 Noise conditions

Speech segments were embedded in six-talker babble from the Connected Speech Test (CST; Cox et al., 1987) in the babble noise condition and in steady-state noise that was spectral-shaped to match the babble in the SSN noise condition. Importantly, the six-talker babble (from three male and three female speakers) is unintelligible but perceived as speech from multiple talkers speaking at the same time. Noise was designed as a between-participant factor (i.e., each participant was only assigned to one noise condition). Nineteen participants were tested with the babble noise; twenty-one were tested with SSN.

A pre-test protocol was used to determine a speech-to-noise ratio (SNR) for each participant, aiming for 90–100% intelligibility. We used this approach for three reasons. First, there is significant variability across individuals with respect to speech perception in noise ability (Wendt et al., 2018). Second, using individualized SNRs with high intelligibility helped to ensure sufficient processing of the speech input to reveal potential effects of psycholinguistic factors. Lastly, pupillometry data are significantly affected by task difficulty (Zekveld et al., 2018) and are uninformative if the degree of difficulty is high enough to cause listeners to disengage (Zekveld and Kramer, 2014).

To determine individualized SNRs, speech reception thresholds (SRTs) were first measured using IEEE sentences (Rothauser et al., 1969) recorded from the same talker embedded in CST babble or SSN based on assigned condition. An adaptive paradigm (Plomp and Mimpen, 1979) was employed to find the SRT that would consistently allow a participant to recognize all keywords in a sentence. The mean SRT across participants was −5.25 dB (*SD* = 1.32). Given that the sentences presented during testing were longer and more complex than the IEEE sentences and based on pilot data results, an individualized SNR that was 4 dB above this SRT (with a lower limit of −4 dB SNR) was used for speech perception testing to achieve 90–100% overall intelligibility. This SNR (i.e., the participant’s measured SRT plus 4 dB) was then verified using sentences that are linguistically more complex than the IEEE sentences (Carlson et al., 2001) and therefore more similar to those used in the experiment. Intelligibility data were collected using these more complex sentences based on three sets of five sentences to confirm overall intelligibility between 90–100% for each participant. The mean SNR used for testing was −1.325 dB (*SD* = 1.33).

### 2.3 Testing procedure

#### 2.3.1 Pupillometry paradigm

Pupil dilation data were collected using an Eyelink 1,000 plus eye-tracker in remote mode with head support. The left eye was tracked with a sampling rate of 1,000 Hz. During testing, the participant was seated in a dimly lit double-walled sound booth in front of an LCD monitor with a fixed distance of 58 cm between eye and screen. The color of the screen was set to grey (RGB 128, 128, 128) to avoid outer limits of the range of pupil dilation based on previous data (Shen, 2021). Luminance at eye position was 37 lux.

The experiment was implemented with a customized program using the Eyelink Toolbox (Cornelissen et al., 2002) in MATLAB (The MathWorks Inc., 2022). Auditory stimuli were presented over Sennheiser HD-25 headphones at 65 dBA.

Participants were instructed to look at a red fixation cross in the center of the screen throughout each trial. After 2000 ms of silence, an audio stimulus was played. The sound stimuli were onset-aligned with 500 ms of noise (babble or SSN, based on condition) before the sentences started. There was a retention period of 2000 ms after the stimulus finished playing, as is standard in pupillometry studies (see Section 2.4.2 for further information). After the retention period, a yes-or-no comprehension question appeared at the center of the screen in the same red font color as the fixation cross. Participants were instructed to respond as quickly and as accurately as possible using a button box. Response accuracy and reaction times were recorded by MATLAB. The trial terminated immediately after the button press, and a grey box appeared at the center of the screen. Participants were instructed to rest their eyes while the grey box was on the screen. This resting period lasted for 6,000 ms before the next trial started. Pupil dilation data were recorded continuously throughout the entire session. The data file was tagged with time stamps that were synchronized with each of these visual and auditory events. Participants repeated these procedures across three visits at least one week apart. Each visit took approximately 90 min and contained 4 blocks of 25 sentence pairs.

### 2.4 Data processing and analysis

#### 2.4.1 Speech comprehension accuracy and reaction time

Reaction time data were cleaned by removing trials with reaction times more than three standard deviations above or below each individual’s mean reaction time. One participant was removed from further analysis due to a technical error during testing, resulting in removal of 2% of the data. While we used individualized SNR to control overall intelligibility, the individual SNR and accuracy/reaction time (RT) were weakly correlated (*r* = 0.016, *p* > 0.1 for SNR and accuracy, *r* = 0.088, *p* > 0.1 for SNR and RT).

Accuracy data were analyzed using generalized mixed effects logistic regression with package lme4 (Bates et al., 2014) in R version 4.0.3 (R Core Team, 2021) to examine the effects of noise and context. Following the recommended practice of using mixed effects models (Meteyard and Davies, 2020), a base model (*model 0*) that included only linguistic context (EE, WV, BV) was compared to a model (*model 1*) that added factors of noise (babble vs. SSN) to test the main effect of noise. The interaction between noise and context was added in *model 2*. In all models, we allowed for fixed factors of trial order, visit order, and individual SNR, along with by-participant random intercepts and by-item random intercepts. We also tested all models with by-participant and by-item random slopes, but they failed to converge (Barr et al., 2013). In these cases, we simplified the model by backward selection, removing each of the random slopes until we achieved the final random structure that provided the best balance between power and Type-I error rate (Matuschek et al., 2017). The model comparison results are reported in Table 2.

**Table 2:** Model fit comparison and *p*-values for the effect of noise and context on comprehension accuracy.

Model	Model comparisons			Degrees of freedom	*p-*value
	AIC	Log Likelihood	*x* ^2^		
Model_1_ = Accuracy ~ Context + Visit Order + Trial Order + SNR + (1|subject) + (1|item)	6895.0	−3439.5			
Model_2_ = Accuracy ~ Context + Noise + Visit Order + Trial Order + SNR + (1|subject) + (1|item)	6897.0	−3439.5	0.0162	1	0.8988
Model_3_ = Accuracy ~ Context * Noise + Visit Order + Trial Order + SNR + (1|subject) + (1|item)	6898.7	−3438.3	2.3481	2	0.3091

Reaction time data were analyzed using linear mixed effects models with log-transformed RT data, as this transformation method yields the more normalized residuals than other methods (Lo and Andrews, 2015). The same model structure as those used for accuracy data was used for reaction time data. The model comparison results are reported in Table 3.

**Table 3:** Model fit comparison and *p*-values for the effect of noise and context on log-transformed reaction time (RT).

Model	Model comparisons			Degrees of freedom	*p-*value
	AIC	Log likelihood	*x* ^2^		
Model_1_ = RT ~ Context + Visit Order + Trial Order + SNR + (1|subject) + (1|item)	5827.2	−2904.6			
Model_2_ = RT ~ Context + Noise + Visit Order + Trial Order + SNR (1|subject) + (1|item)	5829.2	−2904.6	0.0132	1	0.9087
Model_3_ = RT ~ Context * Noise + Visit Order + Trial Order + SNR + (1|subject) + (1|item)	5832.6	−2904.3	0.5655	2	0.7537

#### 2.4.2 Pupil response

Pupil dilation data were pre-processed using the GazeR library (Geller et al., 2020) in R (Version 4.0.3). As pupil dilation has been shown to be altered by fixation location (Gagl et al., 2011; Hayes and Petrov, 2016), a center area of the screen was defined by ±8° horizontal and ± 6° vertical to obtain a pupil alteration rate of less than 5% (Hayes and Petrov, 2016). Pupil dilation data were removed from further analysis when fixation location was outside of this area, resulting in the loss of 3% of total data. Using the Eyelink blink detection algorithm, missing pupil dilation data were marked as blinking. De-blinking was implemented by interpolation during the time window between 100 ms before the blink and 100 ms after the blink. The curve was further linearly interpolated and smoothed using a 50-point moving average. The pupil dilation data were downsampled to 10 Hz (by aggregating data every 100 ms) before statistical analysis. To control for the trial-level pupil dilation variability before speech processing, baseline pupil dilation was calculated based on mean pupil dilation recorded during the 1,000 ms period immediately before the onset of audio stimulus. The dependent measure was pupil dilation relative to individuals’ baseline pupil dilation of each trial by subtraction. Prior research has demonstrated that baseline subtraction is preferable to baseline normalization because the shape of the pupil response is not affected by baseline subtraction (e.g., Winn et al., 2018; van Rij et al., 2019).

Prior studies that have collected both pupillometric and behavioral data have selected different windows of interest for measuring pupil dilation. A common approach is to select a window starting 1.3 s after the presentation of a critical word (e.g., Just and Carpenter, 1993; Engelhardt et al., 2010; Häuser et al., 2019). Alternatively, pupil dilation may be measured in a window after stimulus offset to capture information retention and processing during this post-stimulus interval (e.g., Piquado et al., 2010; Ayasse et al., 2021). The stimuli set used in the present study has the advantage of not necessitating a decision between these two approaches. The critical word in every sentence pair is located at the end of the second sentence. Continuing to measure pupil dilation during a post-stimulus window prior to presenting a comprehension question allowed for potential insight into differences in processing difficulty not only during speech perception but also while preparing to give a response to a question based on the speech input.

**Figure 1:**
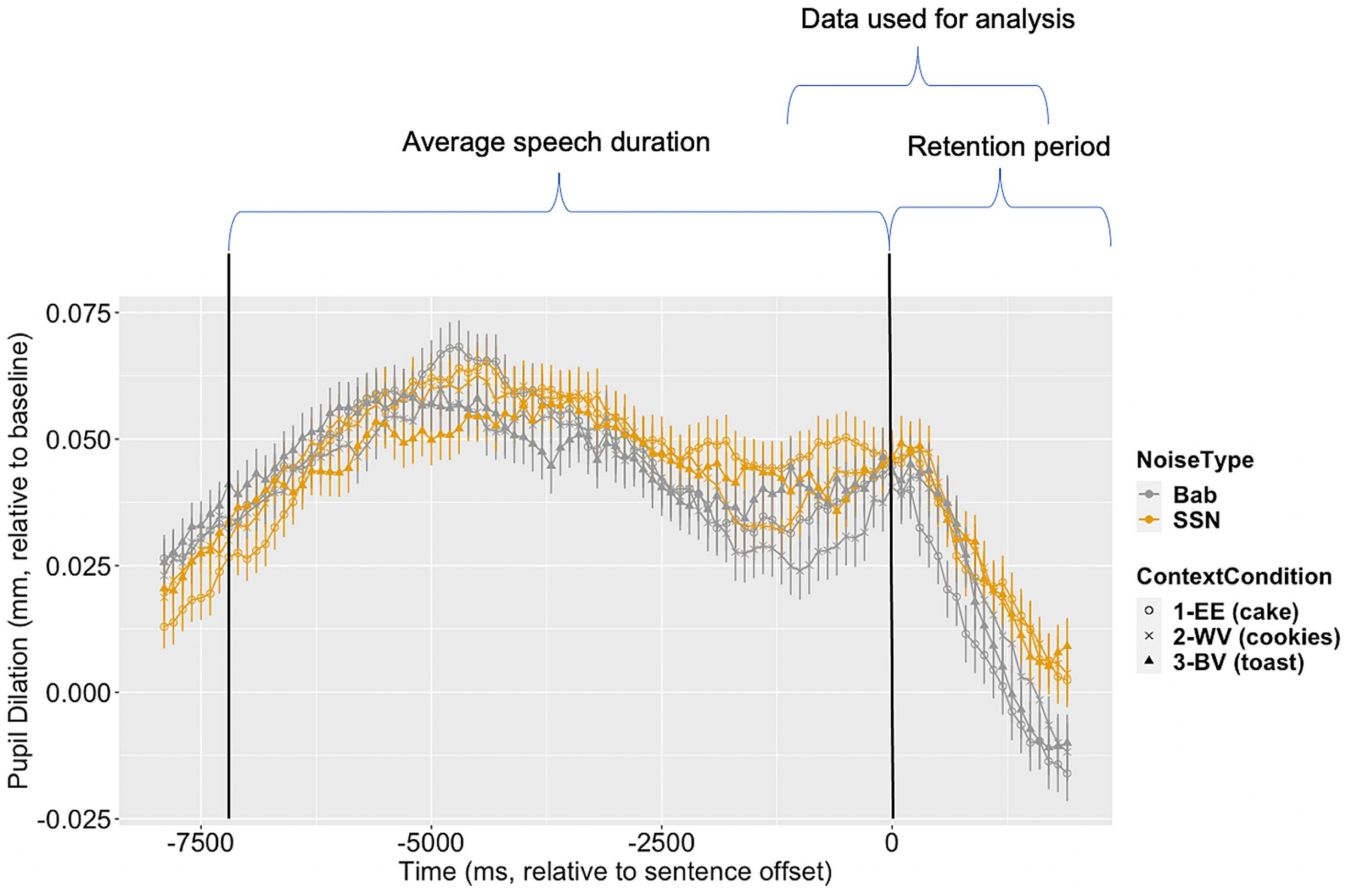
Aggregated pupil dilation relative to baseline (in mm) across the time series (in ms) of stimulus presentation and the post-stimulus retention period prior to comprehension question presentation. Pupil dilation data measured in the babble noise (Bab) condition are pictured in gray, while pupil dilation measured in the Steady-State Noise (SSN) condition are in yellow. Within noise conditions, each context condition is represented by a different shape. The window of time in which pupil dilation data were aggregated for analysis is labeled. Vertical black lines mark sentence pair mean onset and offset. Error bars are ±1 standard error.

To take into account the trajectory of pupil dilation during online processing, PPD data measured during a window of −1,000 to 1,500 ms relative to sentence offset (*M* final word duration = 772 ms) were used for building the linear mixed effects models. Figure 1 illustrates the change pf pupil dilation across time, with sentence duration and analysis window marked. A base model (*model 1*) that only included noise was compared to *model 2*, which included the factor of linguistic context, and *model 3*, which included the interaction between context and noise. In all models, we included variables for trial, visit order, and SNR to control for order and noise level effects. The models were allowed for by-participant and by-item random intercepts. We also tested all models with by-participant and by-item random slopes, but they failed to converge. The model comparison results are reported in Table 4.

**Table 4:** Model fit comparison and *p*-values for the effect of noise and context on peak pupil dilation (PPD).

Model	Model comparisons			Degrees of freedom	*p-*value
	AIC	Log likelihood	*x* ^2^		
Model_1_ = PPD ~ Context + Trial Order + Visit Order + SNR + (1|subject) + (1|item)	4,026,170	−2,013,076			
Model_2_ = PPD ~ Context + Noise + Trial Order + Visit Order + SNR + (1|subject) + (1|item)	4,026,172	−2,013,076	0.2706	1	0.6029
Model_3_ = PPD ~ Context * Noise + Trial Order + Visit Order + SNR + (1|subject) + (1|item)	4,026,161	−2,013,096	15.06	2	< 0.001

## 3 Results

### 3.1 Behavioral data

**Figure 2:**
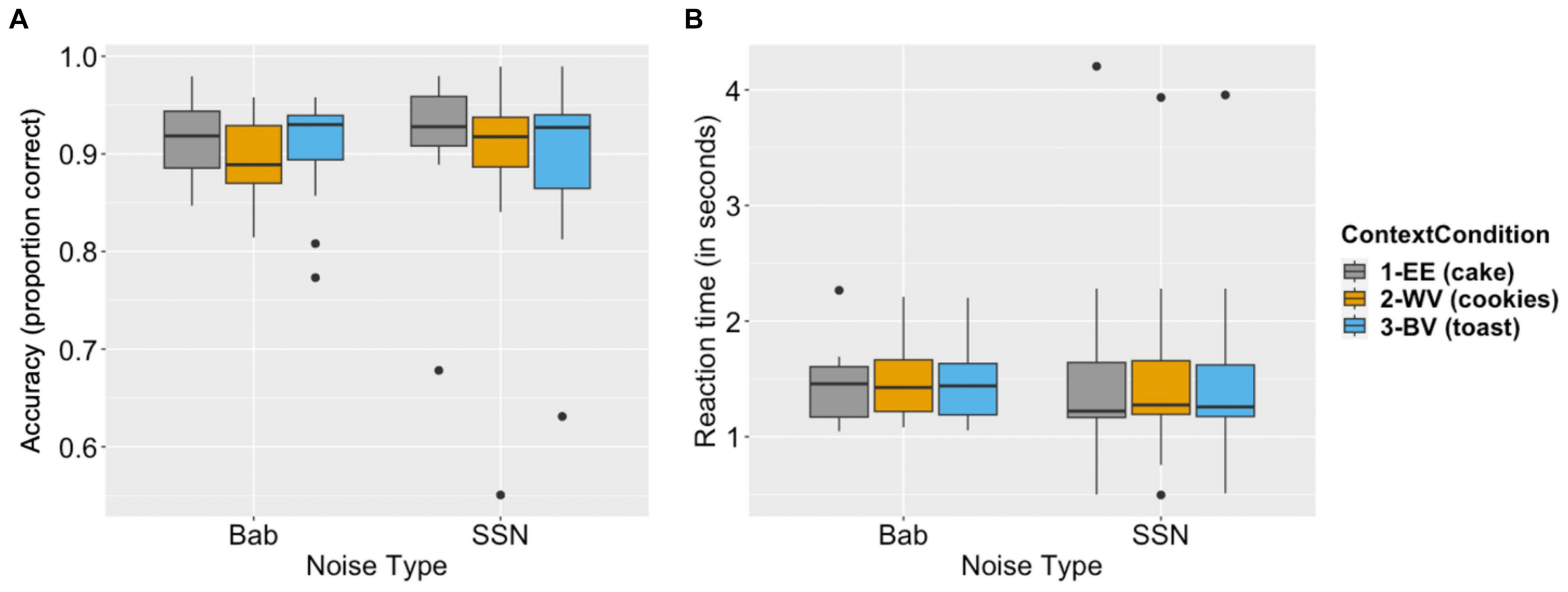
Behavioral data of comprehension accuracy and reaction time. Panel **(A)** depicts the proportion of comprehension questions answered correctly for each context condition (expected exemplar, EE; within-category violation, WV; between-category violation, BV) in the babble noise (Bab) condition and the Steady-State Noise (SSN) condition. Panel **(B)** depicts how much time (in seconds) elapsed before participants provided an answer to post-stimulus comprehension questions for each context condition (EE, WV, BV) in both noise conditions (babble and SSN). The boxes indicate first quartiles, third quartiles, and medians; the whiskers indicate maximum and minimum.

Accuracy and reaction time data are reported in Figure 2.

#### 3.1.1 Comprehension accuracy

Model comparison results showed that neither noise type nor the interaction between context and noise significantly improved model fit (see Table 2 for model comparison results). Using Model 1 as our final model, we found comprehension accuracy was significantly lower in the semantically related but contextually incongruous within-category violation (WV) condition as compared to the expected exemplar (EE) condition (*β* = −0.276, *p* < 0.001). Comprehension accuracy was also lower in the semantically unrelated and contextually incongruous between-category violation (BV) condition than in the EE condition (*β* = −0.178, *p* < 0.05). The results based on the final model are reported in Table 5.

**Table 5:** Final model (Model_1_) for the effect of noise and context on comprehension accuracy.

Fixed effects				
	Beta	SE	*t*-value	*p*
Intercept	2.5543	0.1947	13.114	< 0.001
Within-category Violation (WV)	−0.2767	0.0799	−3.459	< 0.001
Between-category Violation (BV)	−0.1785	0.0812	−2.198	< 0.05
Visit order	0.0073	0.039	0.187	0.851
Trial order	−0.0027	0.0011	−2.48	< 0.05
SNR	0.0368	0.0699	−0.526	0.598

Random effects		
	Variance	S.D.
Item (Intercept)	0.2712	0.5208
Subject (Intercept)	0.2967	0.5447

Final model equation: Accuracy ~ Context + Visit Order + Trial Order + SNR + (1|subject) + (1|item).

#### 3.1.2 Reaction time

Neither the factor of noise type nor the interaction between context and noise significantly improved model fit (see Table 3 for model comparison results). Using Model 1 as our final model, comprehension reaction time was not significantly different when comparing the WV and BV conditions to the EE condition (WV: *β* = 0.007, *p* = 0.30, BV: *β* = 0.006, *p* = 0.38). The results based on the final model are reported in Table 6.

**Table 6:** Final model (Model_1_) for the effect of noise and context on log-transformed reaction time (RT).

Fixed effects				
	Beta	SE	*t*-value	*p*
Intercept	0.4438	0.0725	6.114	< 0.001
Within-category Violation (WV)	0.0076	0.0075	1.023	0.306
Between-category Violation (BV)	0.0060	0.0075	0.878	0.380
Visit order	−0.0601	0.0038	−16.082	< 0.001
Trial order	−0.0004	0.0001	−4.413	< 0.001
SNR	0.0237	0.0373	0.636	0.529

Random effects		
	Variance	S.D.
Item (Intercept)	0.0064	0.0800
Subject (Intercept)	0.0985	0.3139

Final model equation: RT ~ Context + Visit Order + Trial Order + SNR + (1|subject) + (1|item).

### 3.2 Peak pupil dilation (PPD)

**Figure 3:**
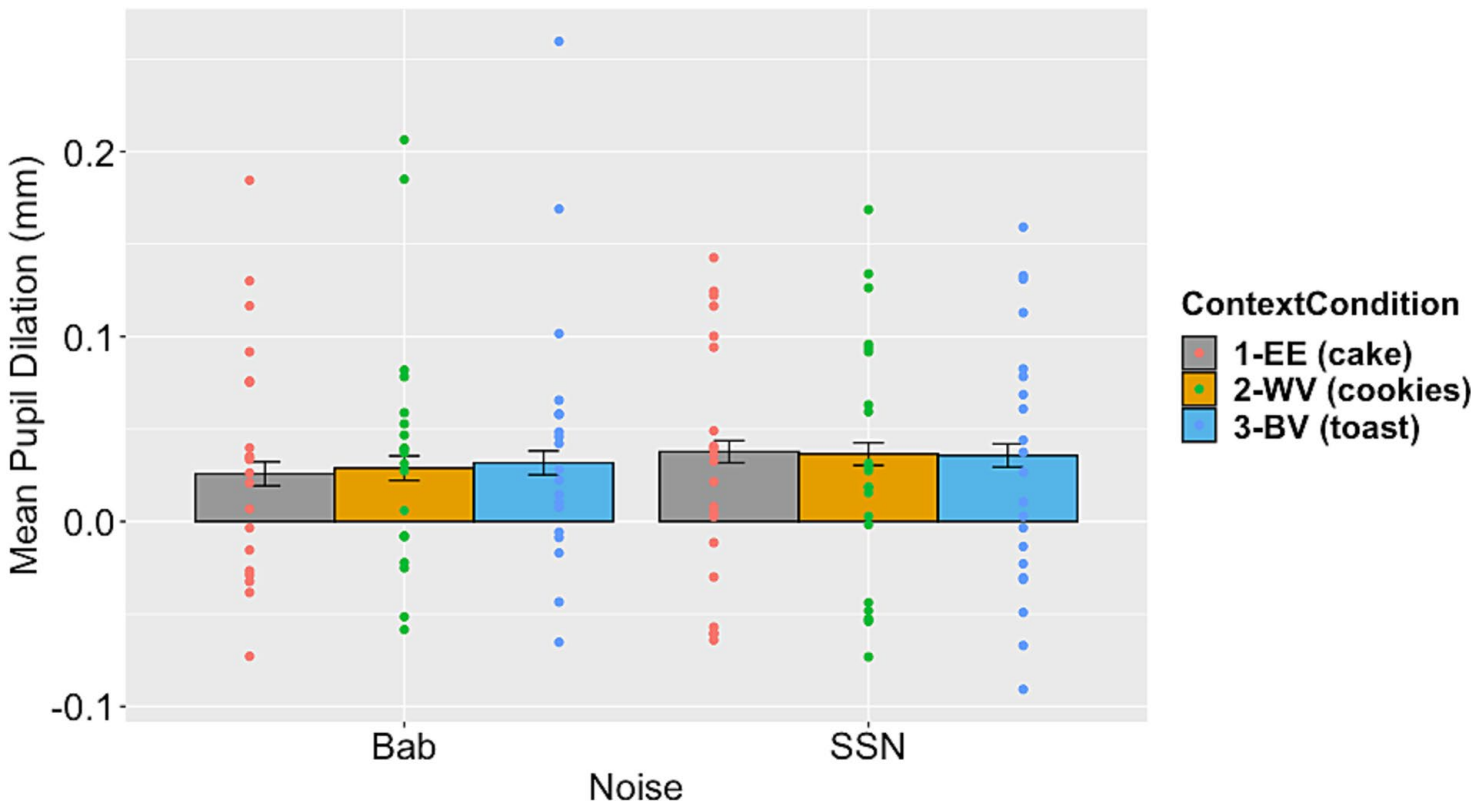
Mean peak pupil dilation measured within the defined window of interest (−1000 to +1500 ms relative to speech offset) for both noise conditions by context condition. Individual participant data is overlayed. Error bars are ±1 standard error.

For the PPD data, while the factor of noise type did not significantly contribute to the model fit, the interaction between context and noise type did (see Table 4 for model comparison results). Moving forward with Model 3 as our final model, it showed a significant interaction between linguistic context and noise condition (see Table 7 for Model 3 results). Figure 3 illustrates the mean PPD across context and noise conditions.

**Table 7:** Final model (Model_3_) for the effect of noise and context on peak pupil dilation (PPD).

Fixed effects				
	Beta	SE	*t*-value	*p*
Intercept	155.4	33.94	3.867	< 0.001
Within-category Violation (WV)	5.302	2.476	2.141	< 0.05
Between-category Violation (BV)	11.70	2.485	4.707	<0.001
Noise type	25.74	51.19	0.503	0.618
WV x Noise	9.346	4.952	1.888	0.059
BV x Noise	19.27	4.967	3.88	< 0.001
Trial order	−1.433	0.035	−40.596	< 0.001
Visit order	−11.68	1.259	−9.272	< 0.001
SNR	−10.44	19.22	−0.543	0.59

Random effects		
	Variance	S.D.
Item (Intercept)	799.6	28.28
Subject (Intercept)	19,663.4	140.23

Final model equation: PPD ~ Context * Noise + Trial Order + Visit Order + SNR + (1|subject) + (1|item).

To reveal the detailed comparisons in the significant interaction, the estimated marginal means and contrasts were calculated using the “emmeans” package in R (Lenth et al., 2023), allowing us to examine the differences between the context conditions in both of the noise conditions. The overall contrast between the two noise conditions was not significant (Est. contrast = −25.7, *p* > 0.1). In the babble condition, PPD was higher in the WV and BV conditions compared to the EE context condition (WV vs. EE: Est. contrast = −9.975, *p* = 0.06; BV vs. EE: Est. contrast = −21.336, *p* < 0.0001). This result indicates that listening was more effortful in the semantically incongruous conditions (WV and BV) than in the congruous condition (EE) in babble. In the SSN condition, PPD was not significantly different between WV and EE, or the BV and EE conditions (WV vs. EE: Est. contrast = −0.629, *p* > 0.1; BV vs. EE: Est. contrast = −2.061, *p* > 0.1). This result indicates that listening effort was similar in the semantically congruous (EE) condition compared to the semantically incongruous (WV and BV) conditions in SSN.

## 4 Discussion

The first aim of this study was to compare the effects of unintelligible multi-talker babble noise to SSN on the perception of complex linguistic input. The second aim was to determine whether perceptual complexity and linguistic context interact during the perception of complex linguistic input in different noise conditions. We used both offline behavioral and online physiological outcome measures to elucidate the individual and interactive effects of perceptual and linguistic complexity on speech perception in noise.

### 4.1 Effects of context and noise

Consistent with prior psycholinguistic research, we observed a main effect of linguistic context in the accuracy data. This finding extends existing literature (e.g., Kutas, 1993; Federmeier et al., 2002; Delong et al., 2011) by demonstrating the linguistic context effect during speech perception in background noise. As demonstrated by the behavioral data, the WV and BV context conditions were more challenging than the EE condition. This result replicates the findings from Federmeier et al. (2002) and provides evidence that the linguistic context facilitated processing of sentences with an EE cloze. Interestingly, the effects were stronger when comparing the EE condition to WV (i.e., the condition with a final word that is contextually incongruous but semantically related to the expected exemplar) than when comparing the EE condition to BV (i.e., the condition with the incongruous and unrelated cloze). These results in the behavioral data may be explained by a possible strategy that participants adopted when answering the comprehension questions. Given that none of the questions were related to the cloze and the speech intelligibility was high, listeners may have ignored the incongruous and unrelated cloze in the BV condition. The cloze in the WV condition, however, may have been more difficult to ignore because of its semantic relationship to the expected cloze. Considering the evidence that upcoming information is pre-activated during predictive processing (e.g., Kuperberg and Jaeger, 2016), pre-activation of the EE cloze may have also resulted in some activation of the semantically related WV cloze based on the spreading activation model of semantic processing (Collins and Loftus, 1975). This semantic activation may have made employing the strategy of ignoring incongruous final words more difficult for the semantically related WV clozes compared to the unrelated BV clozes.

A main effect of noise type was not observed based on accuracy, reaction time, or listening effort. This null effect suggests that the acoustic-phonetic interference from speech babble compared to steady state noise does not have a significant impact on the speech comprehension process. Based on the literature related to effortful listening (e.g., Rabbitt, 1991), we hypothesized that even with perfectly intelligible speech, comprehension may still be affected by the perceptual interference from noise, as comprehension necessitates greater depth of processing than intelligibility (Chapman and Hallowell, 2021; Beechey, 2022). We therefore used individualized SNRs that allowed for high levels of intelligibility in order to isolate and examine the effects of this type of informational masking on comprehension. While we chose this noise comparison based on prior literature (e.g., Hoen et al., 2007; Rosen et al., 2013; Wendt et al., 2018), this lack of effect may be a result of using a fairly favorable individualized SNR for a group of younger adults with normal hearing.

The low task demands associated with the comprehension task could also have contributed to the null effect of noise. None of the questions directly related to the cloze, and there was a window of time before questions were presented during which participants could continue processing the speech input, making the task easier. With the overall comprehension accuracy above 90%, this task resulted in a resource-limited process (Norman and Bobrow, 1975) in which the task demands were within the limit of the resource and increases in task demand did not lead to decreases in performance. Given that the noise levels used in this study (SNR between −4 and 2 dB) are fairly close those positive SNRs found in many natural listening environments (Smeds et al., 2015; Wu et al., 2018), the perceptual complexity in babble in real life communication may not significantly affect speech processing for young adults with normal hearing. Whether this result holds with a more challenging behavioral task or for older adults with hearing loss (who are known to have more difficulty with speech understanding in noise) remain questions for future research.

### 4.2 Interaction between noise and context

The second objective of this study was to examine the potential interaction between linguistic context and noise. We observed an interaction between the type of background noise and the linguistic context in pupil dilation data. In the babble noise condition, mean PPD in the window of interest surrounding item offset was largest in the BV context condition and smallest in the EE context condition, while we observed a different pattern in the SSN noise condition. These findings demonstrate that speech processing under perceptually challenging conditions (i.e., babble) is less effortful with facilitative linguistic context. In other words, listeners make use of linguistic context to resolve perceptual ambiguity that may arise in acoustically adverse scenarios. This result provides support for the hypothesis that with greater perceptual complexity (i.e., in the babble noise condition), participants were more inclined to use the linguistic context. They expended less listening effort (evidenced by lower PPD) when the final word of a sentence pair aligned with expectations based on the linguistic context (i.e., in the EE context condition) compared to contextual violations (i.e., in the WV and BV conditions). Conversely, listening effort was not significantly different across context conditions in SSN. This result suggests that lower perceptual complexity in conjunction with low task demands allowed participants to employ listening strategies based on the expectation of an incongruous cloze. These possibilities are explored in more detail below.

The results in babble noise extend existing accounts of the effects of linguistic and perceptual complexity during online speech processing. As previously noted, the ELU model (Rönnberg et al., 2013) provides an explanation of lexical processing in noise, but does not capture linguistic processing beyond the word recognition stage. Pupillometry research providing support for this framework has also largely focused on the lexical level. For example, in a study examining the effects of SNR and lexical competition in isolated word recognition, pupil responses were greater in both magnitude and duration with less favorable SNRs and increased lexical competition, reflecting greater listener effort (Kuchinsky et al., 2013). Similarly, Wagner et al. (2016) examined the effects of speech signal degradation on lexical selection and found that listening to degraded speech made selection among multiple lexical competitors more effortful and, crucially, more time consuming. This increase in effort and duration at the lexical selection stage resulting from perceptual ambiguity may also have effects on later stages of processing, as suggested by our findings. Disruptions in identification of individual lexical items at the recognition stage could impact the typical time course of speech processing, leading to difficulty and delays at the comprehension stage, when sentence-and discourse-level processing occurs (Pichora-Fuller et al., 2016; Gordon-Salant et al., 2020). These delays in word recognition and speech comprehension could in turn hinder the completion of a coherent mental representation (i.e., situation model) of the overall meaning of an utterance when an incongruous word is encountered.

An important feature of this study is the use of speech material that provided context and allowed participants to build situation models, as ERP studies of violations of lexical predictions based on semantic context during reading found distinct patterns in brain responses to low-versus high-context input (Brothers et al., 2020). The use of longer, more complex speech material and a comprehension task allowed us to examine the effects of these delays on processing. The results in babble noise may reflect compounding effects of initial delays across stages of processing. This perceptual challenge cost appeared to be magnified and modulated by linguistic difficulty, based on the interaction of noise and context. The pupillometric results may indicate that with increased perceptual complexity, participants were less confident in the situation model they were constructing during listening, and the model fell apart at the final word when it did not fit the preceding context. Increased PPD in the condition with greater acoustic (i.e., babble noise) and linguistic complexity could indicate that participants expended more cognitive effort to complete the situation model they had been building based on the input once they were presented with a contextually incongruous final word. This finding supports the prediction that informational masking pushes listeners toward greater reliance on linguistic context. The alternative prediction that informational masking would deplete cognitive resources needed to use contextual information is not supported by these results based on the differences across context conditions in babble noise.

In the perceptually favorable SSN condition, there was no significant effect of context on PPD. This result is in contrast to the results in the babble condition. One possible explanation for this result relates to individual differences in engagement with the speech material across noise conditions, in combination with the use of a listening strategy in the SSN condition. Recent findings have highlighted nuances in pupil response based on task demands and listener engagement. For example, pupil response was larger during an intelligibility task when participants listened to clear speech compared to casual speech in both quiet and noise (Mechtenberg et al., 2023). This finding is counterintuitive given that listening to clear speech is less effortful than listening to casual speech (Zekveld et al., 2014). However, the participants in the Mechtenberg et al. (2023) study may have been able to more greatly engage with the easier clear speech stimuli in order to optimize performance. The results of the present study may reflect a similar type of strategy: participants had greater engagement with the sentences presented in the less perceptually complex SSN compared to the more complex babble noise. With this stronger engagement during processing, participants may have been able to more efficiently generate predictions about the expected cloze, and according to expectation-based theories of language processing (e.g., Hale, 2001; Jurafsky, 2003; Levy, 2008). As a result, the lower perceptual challenge in SSN may have enabled participants to anticipate the final word of the sentence pair before hearing it, allowing them to finish processing the speech input in advance of the presentation of the cloze. Further, with this prediction and pre-processing, participants may have been able to employ a strategy for completing the experimental task with greater efficiency and less listening effort.

Only one third of the experimental trials had a cloze that fit the linguistic context. Listeners are able to adapt to speakers who are known to be unreliable (e.g., Brothers et al., 2019), to have a communication disorder (e.g., Ward and Mack, 2022), or to have “non-native” speaker status (e.g., Bosker et al., 2019). Additionally, recent ERP research indicates that task-related goals affect the generation of lexical predictions (Brothers et al., 2017). In the current study, participants in the SSN condition who were posed with a lower perceptual challenge than participants in the babble noise condition may have adopted a strategy of ignoring the incongruous clozes in the WV and BV conditions and base their situation model on the predicted, pre-processed final word. On this account, similar PPD across the context conditions in SSN reflects participants’ ability to employ a more efficient listening strategy (i.e., ignoring incongruous final words).

Another recent study combining EEG and pupillometry to examine context effects in noise (Silcox and Payne, 2021) provides additional support for the possibility that the similarity in PPD across context conditions in SSN is due to listening strategies adopted by participants in this less perceptually complex noise condition. The overall results of the Silcox and Payne (2021) study indicated that N400s (brain wave responses to unexpected linguistic input) were smaller when stimuli were presented in noise versus in quiet. However, within the group who listened to sentences in noise, larger N400s occurred for participants who had greater pupil dilation (Silcox and Payne, 2021). This finding suggests that participants were able to use the semantic context to make predictions (i.e., engage in deeper processing) when they expended more listening effort. In the current study, participants’ increased engagement with the less perceptually challenging SSN condition may have resulted in the ability to more efficiently make use of the semantic context to generate predictions. This increased efficiency with which participants were able to process (and pre-process) in SSN as compared to babble noise allowed for adaptation to the task and adoption of a listening strategy, providing support for theories from cognitive science and psycholinguistics regarding pressures toward communicative efficiency (e.g., Gibson et al., 2019).

### 4.3 Limitations and future directions

One factor that could contribute to both the null effect of noise and the non-significant effects of context in SSN is that we used a between-participant design for the noise conditions. In other words, two different groups of young participants were tested across the babble and SSN conditions. It is possible that the individual differences between participants in different noise conditions made them respond to the noise manipulation in different ways. We know individual characteristics affect pupil response during naturalistic language processing. Therefore, the individual variability was likely magnified by the comprehension task, as it requires more in-depth processing than an intelligibility task (Beechey, 2022). Prior research has also suggested that listener motivation (Peelle, 2018), working memory capacity (Miller et al., 2019), and attentional allocation and fluctuation (Unsworth and Robison, 2015) may play a role in pupil response. Future research that compares data across noise conditions using a within-participant design and includes a quiet condition could shed light on the influence of individual variability with respect to these factors.

Future research should also examine the potential effects of cognitive abilities, hearing loss, and aging on the interaction between perceptual and linguistic complexity. Cognitive abilities, particularly inhibition, attention, and working memory capacity, have been linked to speech comprehension in noise ability (e.g., Akeroyd, 2008; Rönnberg et al., 2013; Unsworth and Robison, 2015; Miller et al., 2019; Bhandari et al., 2022). For instance, while the comprehension task in the current study relies heavily on the listeners’ ability to control their attention in order to make predictions based on the context (e.g., Bhandari et al., 2022), it was out of the scope of the current study to examine the role of this cognitive function by specifically manipulating task demands, attention cues, or distractors. Comparing participant performance on the listening task described here to performance on neuropsychological assessments designed to measure inhibition, attentional control, working memory, and other cognitive factors may shed light on the findings presented here. Additionally, differences linked to aging and hearing loss may affect these processes (e.g., Broderick et al., 2021; Harel-Arbeli et al., 2021; Gillis et al., 2023), which should be investigated by future research.

## 5 Conclusion

This study examined how type of noise (informational versus energetic masking) interacts with linguistic context during speech comprehension. There were no effects of noise type on comprehension reaction time or accuracy. Pupillometry data in the energetic masking condition were non-significant, but may reflect the ability to employ a more efficient task-based listening strategy. Results of the informational masking condition were consistent with predictions based on theory and empirical findings from hearing science and psycholinguistics. These findings suggest that listeners depend on linguistic context to support processing in challenging listening environments.

## Data availability statement

The raw data supporting the conclusions of this article will be made available by the authors, without undue reservation.

## Ethics statement

The studies involving humans were approved by the Temple University Institutional Review Board. The studies were conducted in accordance with the local legislation and institutional requirements. The participants provided their written informed consent to participate in this study.

## Author contributions

LF: Conceptualization, Investigation, Writing – original draft, Methodology, Project administration. GD: Conceptualization, Writing – review & editing. JS: Conceptualization, Formal analysis, Investigation, Software, Writing – original draft, Data curation, Funding acquisition, Methodology, Project administration, Resources, Supervision, Visualization.
